# A magnetically actuated, optically sensed tensile testing method for mechanical characterization of soft biological tissues

**DOI:** 10.1126/sciadv.ade2522

**Published:** 2023-01-11

**Authors:** Luca Rosalia, Adrien Hallou, Laurence Cochrane, Thierry Savin

**Affiliations:** ^1^ Health Sciences and Technology Program, Harvard-MIT, Cambridge, MA, USA.; ^2^ Institute for Medical Engineering and Science, MIT, Cambridge, MA, USA.; ^3^ Cavendish Laboratory, Department of Physics, University of Cambridge, Cambridge, UK.; ^4^ Wellcome/Cancer Research UK Gurdon Institute, University of Cambridge, Cambridge, UK.; ^5^ Wellcome/Medical Research Council Stem Cell Institute, University of Cambridge, Cambridge, UK.; ^6^ Department of Engineering, University of Cambridge, Cambridge, UK.

## Abstract

Mechanical properties of soft biological tissues play a critical role in physiology and disease, affecting cell behavior and fate decisions and contributing to tissue development, maintenance, and repair. Limitations of existing tools prevent a comprehensive characterization of soft tissue biomechanics, hindering our understanding of these fundamental processes. Here, we develop an instrument for high-fidelity uniaxial tensile testing of soft biological tissues in controlled environmental conditions, which is based on the closed-loop interaction between an electromagnetic actuator and an optical strain sensor. We first validate the instrument using synthetic elastomers characterized via conventional methods; then, we leverage the proposed device to investigate the mechanical properties of murine esophageal tissue and, individually, of each of its constitutive layers, namely, the epithelial, connective, and muscle tissues. The enhanced reliability of this instrument makes it an ideal platform for future wide-ranging studies of the mechanics of soft biological tissues.

## Teaser

An electromagnetic device enables high-fidelity mechanical characterization of soft biological tissues.

## INTRODUCTION

Mechanical properties of soft tissues, such as stiffness, strength, and viscoelasticity (*1*), are key to numerous biological processes (*2*), including embryonic morphogenesis (*3*–*5*), postnatal development (*6*), tissue homeostasis (*7*, *8*), tissue physiological function (*9*, *10*), and aging (*11*). They are also central to the initiation and progression of several pathologies, from cancer (*12*), wound healing (*13*), and fibrosis (*14*) to cardiovascular diseases such as aneurysm, atherosclerosis, and heart failure (*15*–*18*). Despite their relevance to physiology and disease, available mechanical data on biological tissues are conspicuously sparse due to limitations in existing characterization tools and substantial discrepancies among the numerous mechanical testing methods (*1*, *19*, *20*). For instance, Young’s modulus (*E*) measurements of the human skin via indentation methods yield values of approximately 35.0 kPa, whereas tensile tests generate values in the 100- to 200-kPa range (*21*). Despite being affected by several limitations, indentation with a nanoindenter or an atomic force microscope (AFM) is one of the most popular mechanical testing methods for biological tissues. This method involves measuring the penetration depth of a probe into a given tissue as a function of the load of the indenter. The mechanical properties of the tissue are then indirectly evaluated using arbitrary fitting models, which are specific to the geometry of the indenter and often require knowledge of the Poisson’s ratio of the tissue under investigation (*22*). Further, the load and the resulting elongations that tissue samples are subjected to during AFM testing are typically in the order of piconewtons and micrometers, respectively. In addition, loading is only applied to the superficial layers of the tissue and in the direction perpendicular to the sample. These conditions are only marginally representative of physiological conditions, considerably limiting the relevance of AFM testing to studies of human physiology and disease (*1*).

Uniaxial tensile testing—the gold standard mechanical testing method for engineering materials—provides a way to overcome some of these limitations by allowing for the direct evaluation of mechanical properties of the tissue, such as its Young’s modulus, solely by knowledge of the applied load, the elongation of the sample, and its geometry (*9*, *23*). Current tensile testing instruments for soft tissues are based on customized commercial devices initially designed to test engineering materials (*22*) or developed in-house for the particular tissue sample under investigation (*16*, *23*, *24*). Commercial instruments include the Microtest series (Gatan Inc., CA, USA), the BioTense (ADMET Inc., MA, USA), and the Modular Force Stage (Linkam Scientific Instruments Ltd., UK) devices. Critically, the limited force and strain ranges that can be achieved by these instruments make them largely unsuitable to characterize the broad spectrum of mechanical properties of soft biological tissues. Further, they suffer from low output resolution, are associated with elevated costs, are unable to recreate physiologic environmental conditions of the tissues, and, generally, cannot be integrated with commercial inverted confocal microscopes, preventing imaging studies concurrent to mechanical testing.

The lack of appropriate instruments for tensile testing of soft biological tissues and, consequently, of standardized datasets greatly hinders advances in mechanobiology and their translational applications. To overcome these limitations, we propose a tensile testing apparatus that is capable of measuring the mechanical properties of small tissue samples in physiologically relevant conditions. Our device relies on magnetic actuation and optical sensing, enabling simultaneous measurements of the force applied to the tissue and its elongation. The force is due to the interaction between an electromagnet and a ferromagnetic bead attached to the sample, whereas the elongation is tracked by an optical sensor. In this work, we first introduce the design and development of our proposed tensile testing apparatus; then, we validate its performances against conventional tensile testing methods using synthetic elastomers with known mechanical properties. Last, we conduct a study on the biomechanics of the murine esophagus and of its constitutive layers, providing a direct assessment of the performance of our device and demonstrating the high reliability of our method.

## RESULTS

### Design considerations of a tensile testing apparatus for soft biological tissues

One of the primary advantages of our proposed instrument is its ability to enable biomechanical testing at the millimeter scale. This scale corresponds to the typical dimensions of human tissue samples routinely biopsied in clinical settings, as well as to murine embryonic and adult tissue samples, often collected for biomedical research. Millimetric-sized samples are generally smaller than the scale of structural variations due to tissue heterogeneity; however, they are large enough to exhibit bulk mechanical properties. To estimate the force required by our instrument, we assumed that biopsy samples range between *d* = 1.0 to 2.0 mm in diameter and 5.0 to 7.0 mm in length. Given that, under physiologic loading conditions, the stiffness of soft biological tissues ranges from approximately *E* = 0.1 kPa (brain tissue) to *E* = 25 MPa (ligament tissue) (*1*), we computed the required tensile force ($k=\frac{\pi d^{2}}{4}E$) to be ranging from 80.0 μN to over 20.0 N in the elastic regime. Further, assuming that most soft tissues experience an ultimate tensile strength and maximum elongations of around 20.0 MPa and 150%, respectively (*9*), we determined that our instrument must be able to (i) generate mechanical forces ranging from approximately 10^−6^ to 1 N, (ii) enable stretching of the sample over a range of 15.0 mm, and (iii) allow for submillimetric spatial resolution.

**Fig. 1.**
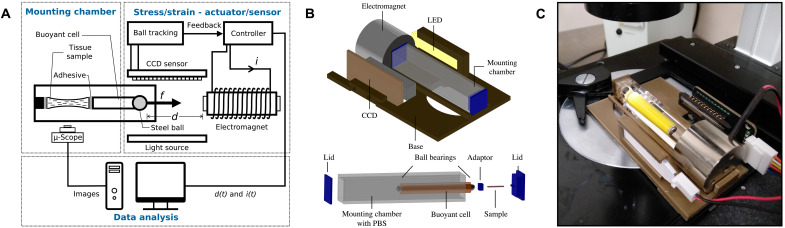
Design of an integrated device for magnetically actuated, optically sensed tensile testing of biological tissues. (**A**) Schematic illustrating the operation principle of the device to enable high-fidelity mechanical characterization of soft biological tissues in the millimeter scale. (**B**) 3D model of the setup, showing (top) the electromagnet, CCD sensor, illumination system, and the microscopy-compatible base; (bottom) the mounting chamber, buoyancy cell, soft tissue sample, 3D printed lids, and sample holders. (**C**) Photo of the tensile testing apparatus in operation on the stage of an Olympus CKX41 inverted microscope.

In a previous study, we demonstrated that a simple magnetic force actuator, composed of a permanent magnet and a steel bead attached to a millimetric sample, can be used to generate forces ranging from 10^−6^ to 10^−3^ N over a range of 2.0 to 8.0 mm from the edge of the magnet (*25*). The force exerted by the magnet on the test specimen is highly nonlinear, exponentially increasing as the steel bead approaches the magnet. This unique feature makes magnetic attraction particularly well suited to exert tensile forces on soft tissues, as these tend to exhibit a strain-stiffening behavior, whereby their stiffness increases nonlinearly at higher deformations (*9*). Here, we develop a more versatile, precise, and robust tensile testing apparatus, where the attraction between a ferromagnetic bead attached to the sample and an electromagnet is used to prescribe known displacement and loading conditions to the sample. In our previous work, the deformation of the sample was tracked using video-microscopy (*25*). Now, we implemented an enhanced optical tracking system whereby the elongation of the sample is directly measured by recording the position of the shadow of the ferromagnetic bead on a linear charge-coupled device (CCD) sensor. Further, we developed a closed-loop controller that modulates the current fed into the magnet, allowing a variety of properties to be measured through both static and dynamic tests, including constant strain, constant stress, oscillatory viscoelastic, and fatigue measurements. Other advantages of our device include its large-force operating range and the noncontact nature of the magnetic interaction, reducing friction and mechanical complexity. Thus, the architecture of the proposed instrument can be divided into three sections, relating to sample handling, force application and deformation measurements through an optomagnetic actuator, and test monitoring with image analysis, as shown in Fig. 1A.

### Hardware components, actuation, and sensing

The mechanical design of the instrument aims to align the light source, CCD, electromagnet, and mounting sample chamber in a reliable yet adjustable configuration. The electrical and optical components are integrated with a custom three-dimensional (3D)–printed support, allowing for simultaneous tensile testing and live imaging of small biological tissue specimens (Fig. 1, B and C). The electrical hardware is integrated with two printed circuit boards (PCBs). The main PCB is designed to attach to the headers of the microprocessor board. It contains the electromagnet driver circuit, the CCD circuitry, and connections with the current sensor, the power source, and the electromagnet. The CCD is located on a separate PCB, which routes the appropriate pins of the chip to a six-wire parallel bus connection to the main PCB.

The device includes a magnetic actuator—an electromagnet generating a variable magnetic field acting on a ferromagnetic steel bead connected to the sample—and an optical system composed of a uniform light source casting the shadow of the bead on the CCD during mechanical testing. The CCD has a resolution of δ*x* = 106.0 μm, a precision of 1.6 μm, and a tracking speed of 200 Hz. The pull force *F*_E_ generated by the electromagnet on the bead is dependent on the diameter *d* of the bead, on its distance from the magnet *x*, and on the current supplied *I*, per Eq. 1

$$F_{E}(x,I,d)=F_{0}I^{2}d^{3}\exp\left(-\frac{x}{x_{0}}\right)$$ (1)

where *x* is the distance from the surface of the magnet to the center of the bead, and $F_{0}=6.13\times 10^{6}\;\frac{N}{A^{2}m^{3}}$ and *x*_0_ = 2.258 ×10^−3^ m are constants. The attractive magnetic force is therefore computed by real-time measurements of the electromagnet current and of the distance between the electromagnet and the bead by the CCD. These are interfaced with the microcontroller of the embedded system, which offers serial-over-USB functionality for communication with an external PC, enabling both the definition of the test parameters and data collection during the experiments. The characterization of the magnetic actuator, of the electromagnet driver circuit, and of the CCD-based optical sensor is shown in figs. S1 to S3.

### Recapitulating physiologic environmental conditions in a mounting chamber

One of the most critical requirements for mechanical testing of soft biological tissues is the need to maintain them as closely to their physiological conditions (e.g., temperature, pH, osmolarity, etc.). To this aim, we designed a mounting chamber that allows immersion of the tissue test specimen in a phosphate-buffered saline (PBS) solution or culture medium. To minimize any interference with the optical system, the mounting chamber was chosen to be transparent and of square cross section. We designed a neutrally buoyant cell to minimize any effects due to gravity and guarantee the alignment between the bead and the CCD sensor. We attached the ferromagnetic beads to both ends of the cell to ensure the symmetry of the cell and to minimize the torque acting on the sample. From Eq. 1, the electromagnetic force exerted on the more distal bead from the magnet was computed to be several orders of magnitude smaller than that acting on the more proximal bead and was thus considered negligible. Instead, this bead was used as an attachment point for the sample. The mounting system, including the chamber, the neutrally buoyant cell, the lids, and the adaptor, is shown in Fig. 1 (B and C).

In silico and in vitro analysis (fig. S4) of the forces acting on the buoyancy cell showed that the drag force is negligible compared to the force required for stretching biological specimens at the millimeter scale (*F*_D_≪ 0.050 μN). In agreement with fluid dynamics theory (*26*, *27*), we found that the drag force increased linearly with velocity at small Reynolds numbers and that smaller cross-sectional areas of the mounting chamber yielded a higher flow resistance (fig. S4). We performed linear regression to the experimental data using the least-square method (*R*^2^ = 0.9789) and obtained an expression of the drag force *F*_D_ as a function of velocity *u*. Here, we report the equation obtained for the mounting chamber used in this article

$$F_{D}(u)=1.68\times 10^{-3}u$$ (2)

where *F*_D_ is in N and *u* is in m s^-1^. For values of strain rates used in this work $\left(\dot{\varepsilon }\leq 0.1\;s^{-1}\right)$, the drag force was therefore negligible compared to tensile load applied to the tissue samples.

### Closed-loop feedback design and implementation

We designed and integrated a closed-loop feedback system in our instrument to ensure electromagnetic stability and highly controllable loading conditions under the effects of unknown sample mechanical properties and disturbances, such as external vibrations, sensor noise, and delays (fig. S5). We implemented an adaptive proportional integral derivative (PID)embedded controller. The block diagram is shown in fig. S5E, and the corresponding mathematical model is derived in the Supplementary Materials. To define numerical values for the closed-loop poles of the system and thus the gains of the adaptive PID controller, we simulated the behavior of the system via a SIMULINK (MathWorks, MA, USA) model (fig. S5F). The closed-loop poles were selected to be *p*_1_ = −60, *p*_2,3_ = −6.5 ± *j*. The first pole value allows the integrator to quickly adapt to the unknown force required to stretch the sample, while the complex conjugate pair of poles provides a fast transient response. To maintain electromagnetic stability at elevated sample deformations, we implemented an adaptive controller, whereby the gains are dynamically recalculated to give unchanging closed-loop poles. The controller design and implementation make the device suitable for mechanical characterization of both linear and nonlinear materials. Further, we limited the maximum current and integral gain of the controller to prevent integrator wind-up for samples displaying elevated damping constants (see the Supplementary Materials). Overall, despite some oscillations in the response of the controller observed at the beginning of the test (fig. S5G), the selected poles allow excellent controllability of the system, with errors never exceeding 10%, as confirmed by the results of our experimental validation (fig. S5H).

### Device validation via testing of synthetic materials with standard methods

**Fig. 2.**
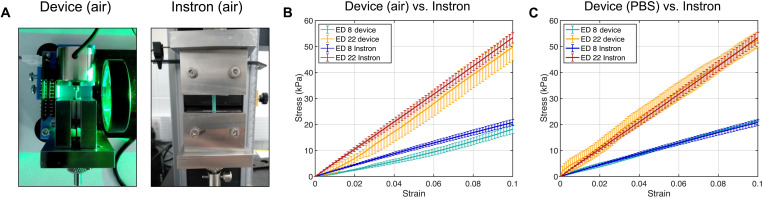
Device validation against standard methods using synthetic material specimens. (**A**) Left: Photo of the device in operation (air configuration), using mechanical grippers to mount the elastomeric specimens. Right: Photo of a 5544 Instron tester with an elastomeric sample mounted using similar grippers. (**B**) Stress-strain curves of the Elite Double 8 and 22 samples on our device (air configuration) and on the Instron tester. (**C**) Stress-strain curves of the Elite Double 8 and 22 samples on our device (PBS in mounting chamber configuration) and on the Instron machine; *n* = 3 samples per test. Error bars represent 1 SD.

We validated our device by evaluating its performance against established methods. To this end, we tested polyvinyl siloxane (PVS) specimens (i.e., Elite Double 8 and 22 formulations, Zhermack, Italy) on our proposed device and on an Instron tensile tester (5544, Instron, MA, USA)—the gold standard for mechanical characterization of engineering materials (Fig. 2A). For this test, we chose PVS as a widely used silicone elastomer with a relatively low stiffness range, which is comparable to that of numerous soft biological tissues. We prescribed displacements up to ε = 0.1 at a constant strain rate $\dot{\varepsilon }\approx 2\times 10^{-3}s^{-1}$. Figure 2 (B and C) illustrates the stress-strain response to 10% strain of the Elite Double 8 (*n* = 3) and 22 (*n* = 3) specimens, as tested on our proposed instrument, either in air or in the mounting chamber filled with PBS, and on the Instron tester. Analysis of the linear elastic behavior of these samples demonstrates the correct functioning of our method, with no statistically significant differences in the Young’s modulus (*E*) when measured on our proposed device or on the Instron tester (Table 1). Further, data show that although testing in the mounting chamber enables the recreation of the physiological environment of biological tissues, the performance of the device remains adequate even when operated in air, allowing for multiple testing configurations.

**Table 1.** Summary of device validation results. Young’s moduli of the Elite Double 8 and 22 PVS specimens measured on our device (PBS, and air configurations) and on the 5544 Instron tester (*n* = 3). For each material, an *F* test showed no statistically significant difference between the Young’s modulus measured on our device and that on the Instron tester.

Method	*E* (kPa)	
	Elite Double 8	Elite Double 22
Device (mounting chamber - PBS)	216.0 ± 11.0	541.8 ± 18.4
Device (no mounting chamber - Air)	213.8 ± 15.6	545.7 ± 35.8
Instron 5544	198.9 ± 10.9	542.8 ± 10.5
*F* test (*P* value)	0.11	0.12

Albeit negligible, in the air configuration, the specimens were observed to exhibit a nonlinear behavior at low strains (Fig. 2B). Using finite element modeling, we showed that nonlinearity can be due to the presence of torque acting on the specimen. This can result from asymmetries in the attachment of the sample on the ferromagnetic bead, which may in turn then lead to mechanical oscillations of the sample (fig. S6, A to C). We mitigated this risk by designing adapters and a support, which facilitate mounting of the sample to our device, minimizing any asymmetries in the attachment (fig. S6D). Further, by characterizing the stress-strain response of an Elite Double 8 specimen during loading and unloading, we showed that the device does not introduce hysteresis in the mechanical behavior of the materials being tested when it is not intrinsically present (fig. S6E).

Last, we investigated the penetration depth of the adhesive used for mounting of the specimens on the device (fig. S6, F and G). Results show that the adhesive penetrates only the surface of the esophageal tissue, with an average penetration depth of 337.93 ± 104.76 μm. These findings suggest that the adhesive-based attachment approach is suitable for testing tissue samples in the millimeter range and has superior spatial resolution than mechanical grippers used by other tensile testing devices (*28*).

### Preconditioning cycles, strain rates, and tissue hysteresis

To demonstrate the ability of our proposed device to characterize the mechanics of biological samples, we studied the mechanical properties of murine esophageal tissue. Propelling food boli from the pharynx to the stomach, the esophagus has a profound biomechanical function (*9*, *10*). Recent work postulated that knowledge of its mechanical behavior from the organ (*29*) to the cellular level is paramount to comprehend its development, homeostasis, physiology, and remodeling during disease (*6*, *30*, *31*).

**Fig. 3.**
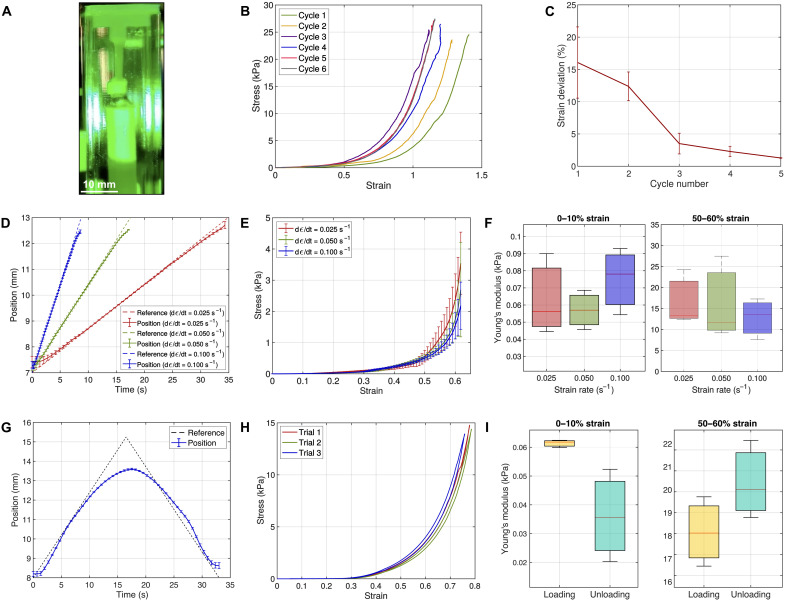
Preconditioning, strain rate, and hysteresis studies of the murine esophagus. (**A**) Lateral view of an esophageal wall specimen under uniaxial tension on the proposed device. (**B**) Representative stress-strain behavior of the esophagus for preconditioning analysis over six loading cycles. (**C**) Absolute strain deviations for each subsequent preconditioning loading cycle (*n* = 3 samples). (**D**) Prescribed reference and measured position signals for strain rate analysis at $\dot{\varepsilon }$ = 0.025, 0.050, and 0.100 s^−1^. (**E**) Stress-strain response at different strain rates. (**F**) Young’s modulus at 0 to 10% and at 50 to 60% elongations for strain rate analysis (*n* = 3 samples). (**G**) Prescribed reference and measured position signals for hysteresis analysis. (**H**) Stress-strain behavior of esophagus samples during loading and unloading cycles for hysteresis analysis. (**I**) Young’s modulus at 0 to 10% and 50 to 60% elongations during the hysteresis cycle for *n* = 1 sample repeated three times. In all graphs, error bars represent 1 SD.

An illustration of a sample of undissected esophageal tissue mounted on our device is shown in Fig. 3A. It should be noted that any torque acting on the specimen must be minimized to ensure correct uniaxial loading conditions. Before investigating the mechanical behavior of the esophagus and its constitutive tissue layers, we studied the effects of preconditioning on measurement repeatability. Preconditioning consists in the repetition of loading cycles and is typically performed on soft biological tissues to obtain a steady and repeatable mechanical response (*23*). Our device allowed us to characterize changes in the stress-strain response (Fig. 3B) and maximum elongation (Fig. 3C) associated with an increasing number of preconditioning cycles. Results demonstrated that variations in the maximum strain dropped below 5% after three preconditioning cycles.

The stress-strain behavior of esophageal wall specimens was measured at three distinct strain rates ($\dot{\varepsilon }$ = 0.025, 0.05, and 0.1 s^−1^). Through this study, we were able to confirm that values of strain rates $\dot{\varepsilon }$ ≤ 0.1 s^−1^ allow us to characterize the mechanical properties of our specimens under quasi-static conditions and therefore assume negligible stress wave propagation during testing (*9*, *32*). The choice of strain rates $\dot{\varepsilon }$ ≤ 0.1 *s*^−1^ conforms with the esophageal biomechanics literature (*33*–*36*). Further, our results show the excellent performance of the control system achieved across the range of strain rates adopted (Fig. 3D), the mechanical response of the esophagus wall (Fig. 3E), alongside the Young’s modulus calculated at both low (0 to 10%) and high (50 to 60%) deformations (Fig. 3F).

We subsequently showed the ability of the device to investigate tissue hysteresis during loading-unloading cycles. We implemented a triangular reference position signal (Fig. 3G) and measured the stress-strain response (Fig. 3H) of the tissue, which we used to extract the Young’s modulus at both low and high strains (Fig. 3I). Tests were carried out at $\dot{\varepsilon }$ = 0.06 s^−1^ and repeated three times on a single specimen. The mechanical response showed only marginal variation between the loading and unloading phases. During unloading, the stress decreased more rapidly than in the loading phase. As a consequence, at elevated strains, the tissue exhibited a slightly higher Young’s modulus during unloading than in the loading phase.

Overall, analysis of the effects of preconditioning, strain-rate variations, and tissue hysteresis demonstrates the versatility of our device and suggests that our proposed method can reliably enable different modalities, configurations, and loading conditions for studies of soft tissue biomechanics.

### Biomechanical characterization of the esophagus as a multilayered soft tissue

**Fig. 4.**
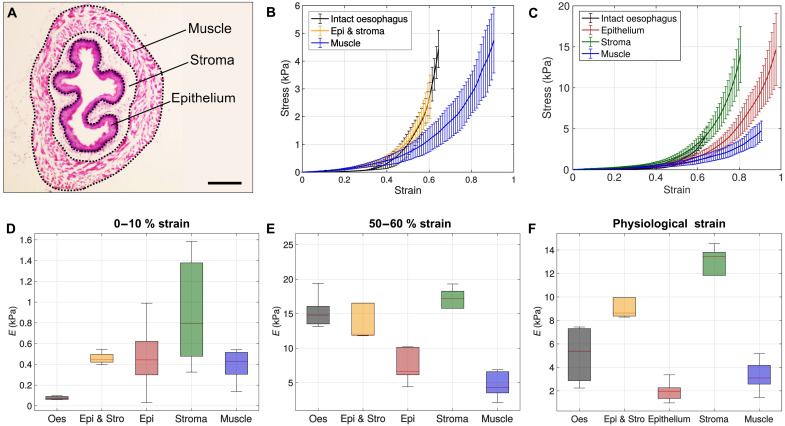
Mechanical characterization of the esophagus as a multilayered tissue. (**A**) Hematoxylin and eosin (H&E)–stained section of the esophagus, showing the epithelial (mucosa), stromal (submucosa), and muscle (tunica muscularis) layers. Scale bar, 200 μm. (**B**) Stress-strain behavior of the intact esophagus, undivided epithelium and stroma, and muscle layer (two-layer model). (**C**) Stress-strain behavior of the intact esophagus and of each individual layer (three-layer model). (**D**) Young’s modulus at 0 to 10% elongations of the intact esophageal wall and of the separated tissue layers. (**E**) Young’s modulus at 50 to 60% elongations of the intact esophagus and of the separated tissue layers. (**F**) Young’s modulus of the intact esophagus and of the separated tissue layers at physiological strains. Three different specimens (*n* = 3) were used for each test and each test was repeated five times. In all graphs, error bars represent 1 SD.

The esophagus is composed of multiple tissue layers surrounding a hollow central lumen, i.e., the mucosa, the submucosa, and the tunica muscularis (Fig. 4) (*37*). The mucosa is constituted of a squamous stratified epithelium with several layers of differentiated suprabasal cells and one layer of self-renewing basal progenitor cells (*6*). The submucosa or stroma contains the lamina propria, underlying the epithelium and consisting mainly of connective tissue, and the muscularis mucosae. The external layer, i.e., the tunica muscularis, is made of the circular inner and the longitudinal outer muscle layers. To date, a comprehensive characterization of the mechanical behavior of the esophagus is missing because of the absence of adequate testing methods and protocols for tissue separation of multilayered biological organs. Therefore, research has been largely limited to opening-angle or inflation testing for measurements of circumferential strains in rats (*38*–*40*), rabbits (*41*, *42*), guinea pigs (*43*), and pigs (*44*–*46*). In this study, we leverage our proposed device to conduct the first uniaxial tensile testing for biomechanical characterization of the entire esophageal tissue, as well as of each of its three main constituting layers.

Figure 4 (B and C) illustrates the stress-strain behavior of the intact esophageal wall and that of the esophagus as a two-layered (i.e., epithelium and stroma, and muscle) and three-layered (i.e., epithelium, stroma, and muscle) tissue, respectively. Results highlight the enhanced reproducibility of the measurements, with errors in the stress-strain response generally below 15%, which constitutes one of the main advantages of this device compared to previous methods. These errors can be mainly attributed to intersample variability, likely due to variations induced by the layer separation processes on a tissue-by-tissue basis. In the two-layer configuration, the inner layer (i.e., epithelium and stroma) exhibited a stiffer behavior than the outer layer (muscle) from elongations approximately ε ≥ 0.45. These findings are in agreement with the literature of inflation testing (*38*). In the three-layer configuration, the stromal layer exhibits the stiffest behavior, dominating the response of the entire esophageal wall. This likely arises from the increasing stiffness exhibited by the stromal tissue following uncrimping of collagen fibers (*47*). Contrarily, we found the muscle layer to be the most compliant, followed by the epithelial and the stroma layers. At low strains (i.e., 0 to 10%), the stroma was found to exhibit the highest modulus, which is only marginally higher than that of the other individual tissue layers (Fig. 4D). At high strains (i.e., 50 to 60%), however, the Young’s modulus of the stroma more distinctly dominates that of the epithelium and of the muscle (Fig. 4E).

Albeit informative, values of the Young’s modulus of the esophageal wall in the intact stress-free state do not provide critical information regarding the mechanical contribution of each layer in physiological conditions, due to the presence of nonzero residual strains. Therefore, for each individual layer, we measured the residual strain in the longitudinal direction (ε_R_) and their thickness *h* (Table 2). Then, we computed the Young’s modulus of the esophageal wall and of each tissue layer at their physiological strain (ε_P_ = ε_R_ + ε_s_), where ε_s_ is the strain prescribed during mechanical testing and ε_R_ is defined above. Findings suggest that, at physiological strains, the response of the intact wall is dominated by the stroma, followed by the muscle and the epithelial layers (Fig. 4F). Last, we validated our results using a semianalytical mathematical model (see Table 2 and the Supplementary Materials) that predicts the behavior of the intact esophageal wall as a two- and three-layer tissue. To our knowledge, these are the first results of the mechanical properties of the esophagus as a three-layer tissue.

**Table 2.** Mechanical characterization of the murine esophageal tissue. Thickness (*h*), longitudinal residual strain (ε_R_), applied longitudinal strain due to tensile testing (ε_S_), longitudinal physiological strain (ε_P_), and calculated Young’s modulus at physiological strain (*E*_P_) of the entire esophagus and of each constitutive tissue layer (*n* = 4 animals with six sections per animal). Strains are computed using measurements of the length of the cut-open esophageal wall as the reference. For the undissected esophageal tissue, experimental measurements are compared with the semi-analytical predictions of the two- and three-layer models.

Tissue	*h* (μm)	ε_R_	ε_S_	ε_P_	*E*_P_ (kPa)
Esophagus (experimental)	312.44 ± 11.44	0.00	0.43 ± 0.06	0.43 ± 0.06	5.11 ± 2.32
Esophagus (two-layer model)	–	–	–	–	5.06 ± 1.04
Esophagus (three-layer model)	–	–	–	–	4.22 ± 0.74
Epithelium and stroma	83.44 ± 5.57	0.06 ± 0.04	–	0.49 ± 0.07	9.68 ± 2.31
Epithelium	45.6 ± 5.53	−0.21 ± 0.08	–	0.22 ± 0.10	1.98 ± 0.82
Stroma	37.84 ± 0.68	0.02 ± 0.12	–	0.45 ± 0.13	12.22 ± 3.05
Muscle	229.03 ± 9.96	−0.02 ± 0.02	–	0.41 ± 0.06	3.26 ± 1.29

## DISCUSSION

In this article, we describe the development of a high-fidelity device for uniaxial tensile testing of soft biological tissues. The device operates as a closed-loop system, generating tensile force by means of the interaction between an electromagnet and a ferromagnetic bead, while optically tracking the displacement of the sample under various loading conditions (Fig. 1A). A mounting chamber enables testing of biological specimens in a controlled environment to mimic their in vivo conditions as closely as possible and preserve their biomechanical properties (Fig. 1B). Following experimental and numerical analysis of the forces acting on the system during testing, we formulated a mechanical model and implemented an embedded control system based on an adaptive PID controller to allow for highly reliable measurements and enable various biomechanical testing modalities.

First, we validated the device by characterizing the elastic properties of synthetic materials, which were shown to be in close agreement with those measured by standard testers for engineering materials (Fig. 2 and Table 1). We then leveraged the proposed apparatus to investigate the biomechanics of the murine esophagus, optimizing the preconditioning protocol, studying mechanical variations associated with varying the strain rate, and characterizing tissue hysteresis due to loading and unloading. We showed that three preconditioning cycles yielded consistent measurements (Fig. 3, B and C), that strain rates $\dot{\varepsilon }$ ≤ 0.1 s^−1^ allowed testing under quasi-static conditions (Fig. 3, D to F), and that the hysteresis response of the tissue could be considered negligible (Fig. 3, G to I). We used the device to conduct the first biomechanical investigation of each individual layer of the murine esophageal tissue, namely, the epithelium, stroma, and muscle (Fig. 4A). Our findings showed that the stroma exhibited the stiffest response at low, high, and physiological strains. The muscle tissue was shown to be less stiff than the epithelium at the stress-free state, while providing a more important mechanical contribution to the esophageal wall than the epithelium at physiological strains (Fig. 4, B to F).

Our study demonstrated the enhanced reliability of the proposed device, which yielded errors in the stress-strain response below 15%. This represents a considerable improvement from a former device developed by this group based on an open-loop permanent magnet, whose measurements were affected by inaccuracies ranging from 20 to 30% (*25*), and establishes a new standard of precision in the field. This demonstrated level of accuracy allowed us to investigate the different mechanical responses of each individual tissue layer of the esophagus in various regimes of force and deformation. Our proposed instrument was shown to successfully address the main limitations associated with other current mechanical testing methods, providing measurements with greater reliability and precision. Therefore, we hope that this device may eventually become the new standard in the field and a unifying tool for biomechanical characterization of biological tissues, resolving the vast heterogeneity in mechanical properties of other tissues and organs.

Future investigations will leverage the controllability of our device further to measure viscoelastic properties of soft biological tissues via creep and stress relaxation experiments, as well as dynamic fatigue testing. Taking advantage of the design of our device, which allows for integration with a confocal or multiphoton microscope, we also endeavor to couple mechanical characterization of soft tissues with live imaging of their extracellular matrix organization using second-harmonic generation (*48*) and of their cellular behavior using genetically encoded fluorescent reporters (*49*). Integrating our device with light-sheet, superresolution microscopy, or a combination thereof could allow us to study, at the molecular level, the real-time response of the nucleus, organelles, cystoskeletal filaments, or even individual transmembrane proteins, ultimately elucidating mechanisms by which cells are able to sense and respond to mechanical stimuli. The current design could be modified to control the temperature, CO_2_/O_2_, and chemical composition of the medium in the mounting chamber, paving the way toward more physiologically relevant investigations of mechanical properties and biological behaviors such as gene expression, cell division, cell fate decisions, tissue growth, and remodeling, in response to both biochemical and mechanical perturbations. Last, in future studies, we aim to investigate the mechanical behavior of a broader range of mouse and human soft tissues, leveraging our device to generate the first “mechanome”—a universal and comprehensive characterization of soft tissues mechanical properties in homeostasis and disease. Through analysis of the biomechanics of healthy tissues and their changes as they occur during disease, our device could eventually identify alterations in tissue properties of diagnostic or prognostic relevance. This could ultimately support physicians in making informed and individualized treatment decisions and contribute to the translation of mechanobiology research into the clinical arena.

## MATERIALS AND METHODS

### Electromagnetic force characterization

A commercial electromagnet (M52173, Eclipse Magnetics, Canada) was used as the actuator of the device. A finite element (FE) model of the magnetic field generated by the coil (fig. S1B) was developed using Finite Element Method Magnetics 4.2 open-source software to evaluate the magnetic force acting on the ferromagnetic bead used in this study. The model performed numerical integration of the Maxwell stress tensor over its volume to generate a set of characteristic curves representing either the force acting on the bead as the function of the current or the force acting on the bead as a function of the distance to the electromagnet (*50*). FE predictions were then validated with experimental measurements of the magnetic force acting on the steel bead using a microbalance with a resolution of 1.0 mg, equating around 10 to 5 N, while the position was adjusted using a micrometer head, with a Vernier caliper scale resolution of 10.0 μm (fig. S1, C and D).

### Optical tracking implementation

Optical tracking of the specimen could be achieved through two different approaches: the first involving the use of an LED, allowing for a compact design and microscope mounting (Fig. 1, B and C), and a second approach that leveraged a combination of a point light source and a positive focal length lens (fig. S3A) to achieve higher spatial resolution. For both approaches, we used a CCD sensor (TCD1304DG, Toshiba, Japan) with an array of 3648 pixels at a distance of 8.0 μm. Using the CCD output (fig. S3B), the resolution of the sensor was computed to be δ*x* = 115.0 μm or 14 pixels for the compact design and δ*x* = 106.0 μm or 13 pixels for the second approach. The small change in optical resolution between the two approaches validated the use of the LED panel design to improve the compactness of the device and integration on a microscope stage.

The driving and readout of the CCD array are directly implemented using the microcontroller unit (MCU) timers, analog-to-digital converters (ADCs), and direct memory access bus (DMA) (fig. S3C). The master clock is implemented using a 16 bit timer, used in pulse width modulation (PWM) mode with a duty cycle of 50%, and set to a period of 21 core clock pulses to generate a 4-MHz square wave from the 84-MHz reference. The integration clear gate (ICG) is implemented with a 16-bit timer in PWM mode and a prescaler of 42 to generate a 2-MHz intermediate base reference. The period is set to 40, leading to a 20-μs CCD integration time. For the ICG, a 32-bit timer was used because of the relatively long period between frame capture events, resulting in a frame rate of 200 Hz. Measurements of the analog output stream of the CCD require synchronous operation of the ADC and automatic handling of the sampled values. This was achieved by a system of four interconnected hardware interrupts, an ADC trigger, and the DMA. The ADC trigger timer was set to 1 MHz, i.e., the CCD data output rate, and generates interrupts that initiate an ADC conversion with 8-bit precision. After the conversion, an interrupt generated by the ADC triggers the DMA to transfer the sampled value to a buffer array, i.e., the same length as the CCD signal. Once the buffer is filled, a DMA Transfer Complete interrupt stops the ADC trigger timer, preventing more samples from being converted, and initiates the execution position measurement algorithm and the next time step of the feedback controller. This cycle is reinitiated by an interrupt generated at the end of an ICG pulse, which restarts the ADC trigger timer at the same time as the next sequence of CCD output data begins, thus continually providing the latest CCD data in the buffer array.

### Controller design

SIMULINK was used to design and simulate the behavior of the PID controller in response to a variety of reference input signals. This allowed us to determine its closed-loop poles and the PID gains. The SIMULINK model is made of six main blocks (fig. S5F). The electromagnet block is governed by the equation for *F*_E_ (Eq. 1) and the transfer function of the MOSFET-based switching circuit, which drives the electromagnet. The sample block describes the Kelvin-Voigt model (*51*, *52*) and the inertia of the ferromagnetic bead. The drag force block is based on the expression for *F*_D_ given in Eq. 2 and allows computation of the net force acting on the sample. Other blocks are the current sensor block, the CCD sensor block, which includes noise, delay, and quantization effect, and the microcontroller block, which was implemented as a MATLAB function block with inputs of the measured current, measured position, and the reference signal and the PWM duty cycle as the output. For the definition of the sample block, stiffness values of *k* ≤ 20 N m^-1^ were used to mimic the behavior of stiff biological tissues at 100% strain. The SIMULINK model and controller design were validated experimentally via testing of Elite Double 8 specimens using a ramp reference position at a rate of $\dot{\varepsilon }$ ≈ 2 × 10^−3^ *s*^−1^ (fig. S5, G and H). Further details of the design and implementation of the controller are provided in the Supplementary Materials.

### Embedded system design

The embedded system is based on the NUCLEO-F103RB board by ST Microelectronics (Switzerland). Its 84-MHz ARM M4 core was used to implement the feedback controller, while its MCU interfaces with the electromagnetic force actuator drive circuit and the CCD sensor. The embedded software (C language) was implemented using the hardware abstraction layer firmware provided by ST Microelectronics. External data acquisition was enabled by a PC virtual terminal using the software PuTTY and a serial link over USB functionality (virtual COM port). The design of the embedded systems is detailed in the Supplementary Materials.

### Component prototyping

The mechanical components of the device, including the base, the buoyancy cell, the mounting chamber lids, the adapters, and the mounting support, were 3D-printed in polylactic acid using an Ultimaker 2+ printer (Ultimaker, The Netherlands) with a layer resolution of 0.1 mm. The buoyancy cell was designed to be a hollow cylinder, with custom dimensions to ensure buoyancy during testing (see the Supplementary Materials). The mounting chamber was manufactured via laser cutting of a clear thin-walled hollow polymethyl methacrylate tube. We determined the length of the chamber (76 mm) to accommodate the buoyancy cell and allow for the desired elongations of the specimen during tensile testing.

### Drag force in vitro and in silico analyses

The drag force *F*_D_ acting on the buoyancy cell in the mounting chamber was evaluated both in vitro and in silico. Experimentally, the cell was first brought to the bottom of a vertically rotated mounting chamber by means of a permanent magnet and then released to float. Analysis was repeated for two sizes of the mounting chamber to investigate the effect of different cross-sectional areas. The drag force was calculated given the knowledge of the inertia and of the volume of the body, and from position measurements obtained by a high-speed camera and of Tracker 5.0 motion analysis software. A fluid-structure interaction model was implemented on Abaqus (Dassault Systèmes, France) to validate the experimental results. In this model, nonzero velocities were defined for the neutrally buoyant object, and no-slip boundary conditions were imposed at the walls of the medium to compute the forces acting in the direction opposite to motion.

### Device validation using synthetic samples

The Elite Double 8 and Elite Double 22 formulations were used to make the PVS samples for validation of our device. The base and the catalyst were manually mixed at a 1:1 volume ratio for approximately 1 min and cured at room temperature in 3D-printed dumbbell-shaped molds and glass capillary tubes (*d*_in_ = 0.58 mm) for testing on the Instron 5544 machine and on our device, respectively. In both cases, we ensured that the length-to-width ratio of each specimen was greater than 4:1 to minimize any shear effects during tensile testing.

During testing on our device, a low current was first applied to ensure the correct alignment of the specimen with the CCD sensor. The sample was then stretched to a minimum of 10% strain to capture the low-strain elastic behavior of the Elite Double specimens. Strain rates $\dot{\varepsilon }$ ≤ 0.1 s^−1^ were used for the Elite Double 8 (*n* = 3) and 22 (*n* = 3) specimens. Each test was repeated three times. The tensile force *F*_E_ applied on the sample was calculated per Eq. 1, and the engineering stress σ and the engineering strain ε were computed as σ = $\frac{F}{A}$ and ε = $\frac{l-l_{0}}{l_{0}}$, where *A* is the cross-sectional area of the sample, and *l* and *l*_0_ are the final and initial gauge lengths, respectively. Testing of the three dumbbell-shaped specimens was carried out on the Instron machine under the same loading conditions using a 50.0-N load cell. The Young’s modulus was computed in the 5 to 10% strain region to minimize artifacts due to stress concentrations induced by mechanical grippers (on the Instron machine and on the device without the mounting chamber) (*28*) and to ensure a fair comparison across all groups. *F* tests were conducted on MATLAB R2020a (MathWorks, MA, USA) to determine statistical significance (*P* < 0.05) of the three groups, i.e., device (PBS), device (air), and Instron.

An FE model was developed to simulate the stress-strain behavior of PVS samples in the presence or absence of torque. Samples were modeled as perfectly elastic cylinders of diameter *d* = 0.58 mm and of length *l*_0_ = 0.3 mm. Simulations were carried out for three different twist angles (ϕ = 0°, 22.5°, and 45°), before applying uniaxial tension to 10% strain. Eight-node brick elements with reduced integration (C3D8R) were used for meshing. The von Mises stress σ_v_ was calculated at the midplane section of the mesh.

### Preparation, testing, and modeling of biological samples

All animal experiments were approved by the local ethical review committee and conducted according to Home Office project license PP0822646 at the Wellcome–CRUK Gurdon Institute of Cambridge University. A total of 12 wild-type C57BL/6J mice (Charles River; strain code 632) of approximately 12 weeks of age were used for this study. All animals were housed between 19° and 23°C, 45 and 65% humidity, and a 12-hour day/12-hour night light cycle. All experiments comprised male and female mice, with no sex-specific differences.

The esophagi were dissected from the body of the animal and cut open. The muscle layer was mechanically separated from the stroma and the epithelium under a dissection microscope using watchmaker forceps. Separation of the epithelial and stromal layers required a 2-hour incubation in PBS/100 mM EDTA at 37°C on an orbital shaker followed by mechanical separation under a dissection microscope using watchmaker forceps. All tissues were subsequently stored in PBS on ice (4°C) and tested within 6 hours on our device at room temperature (21°C). The current literature reports no alterations in mechanical properties of biological tissues induced by EDTA other than on bone tissue (*53*). Rectangular specimens were obtained via longitudinal cuts of the middle esophagus (*l*_0_ = 8.0 mm) (*54*). The sample dimensions were measured under a dissecting microscope fitted with a calibrated eyepiece reticle. The absolute and relative thickness of the different tissue layers of the esophagus were evaluated using esophageal transversal tissue cryosections (7.0 to 10.0 μm) stained with hematoxylin and eosin (*n* = 4 animals with six sections per animal) and imaged using an EVOS XL Core Imaging System (Thermo Fisher Scientific, MA, USA).

A mounting support was used to guarantee the alignment of the wet tissue specimen during gluing with approximately 1 μl of cyanoacrylate (Loctite, OH, USA) to the chamber lids at one end and to the bead-to-specimen adaptor at the other end (fig. S6D). Mechanical testing was conducted under quasi-static conditions ($\dot{\varepsilon }$ = 0.025 s^−1^) on three different specimens per test (*n* = 3), and each test was repeated five times. A multilayer mechanical model of composite materials (*55*) was used to evaluate the contribution to esophageal mechanics of each individual tissue layer and validate our experimental results (see the Supplementary Materials).

We evaluated the penetration of the cyanoacrylate adhesive into nine esophageal tissue specimens (*n* = 9). After separation of the muscle layers, the epithelium-stroma tissue composites were laid flat on top of a #1 glass coverslip and kept wet with PBS. Using a cut micropipette tip, we added 1 μl of cyanoacrylate glue to replicate the gluing protocol used during tensile testing. The adhesive was then left to set for 15 min before images of the sample were acquired using a stereomicroscope with oblique illumination. Images were segmented using the machine learning pixel classification software ilastik (*56*) to obtain semantic segmentation masks of the tissue samples and of the glue-imbibed tissue regions. For each sample, the penetration depth was measured in six distinct locations.

## Notes

View/request a protocol for this paper from *Bio-protocol*.
