# Supplementary material for: A magnetically actuated, optically sensed tensile testing method for mechanical characterization of soft biological tissues
Source: Sci Adv. 2023 Jan 11;9(2):eade2522. doi: 10.1126/sciadv.ade2522 (PMC9833656; doi:10.1126/sciadv.ade2522)
Supplement: Supplementary file 1 — Figs. S1 and S6 Supplementary Information [file sciadv.ade2522_sm.pdf]

Science Advances
AAAS

Supplementary Materials for

A magnetically actuated, optically sensed tensile testing method for mechanical characterization of soft biological tissues

Luca Rosalia *et al.*

Corresponding author: Thierry Savin, t.savin@eng.cam.ac.uk

Sci. Adv. **9**, eade2522 (2023)
DOI: 10.1126/sciadv.ade2522

This PDF file includes:

Figs. S1 and S6
Supplementary Information

Supplementary Figures

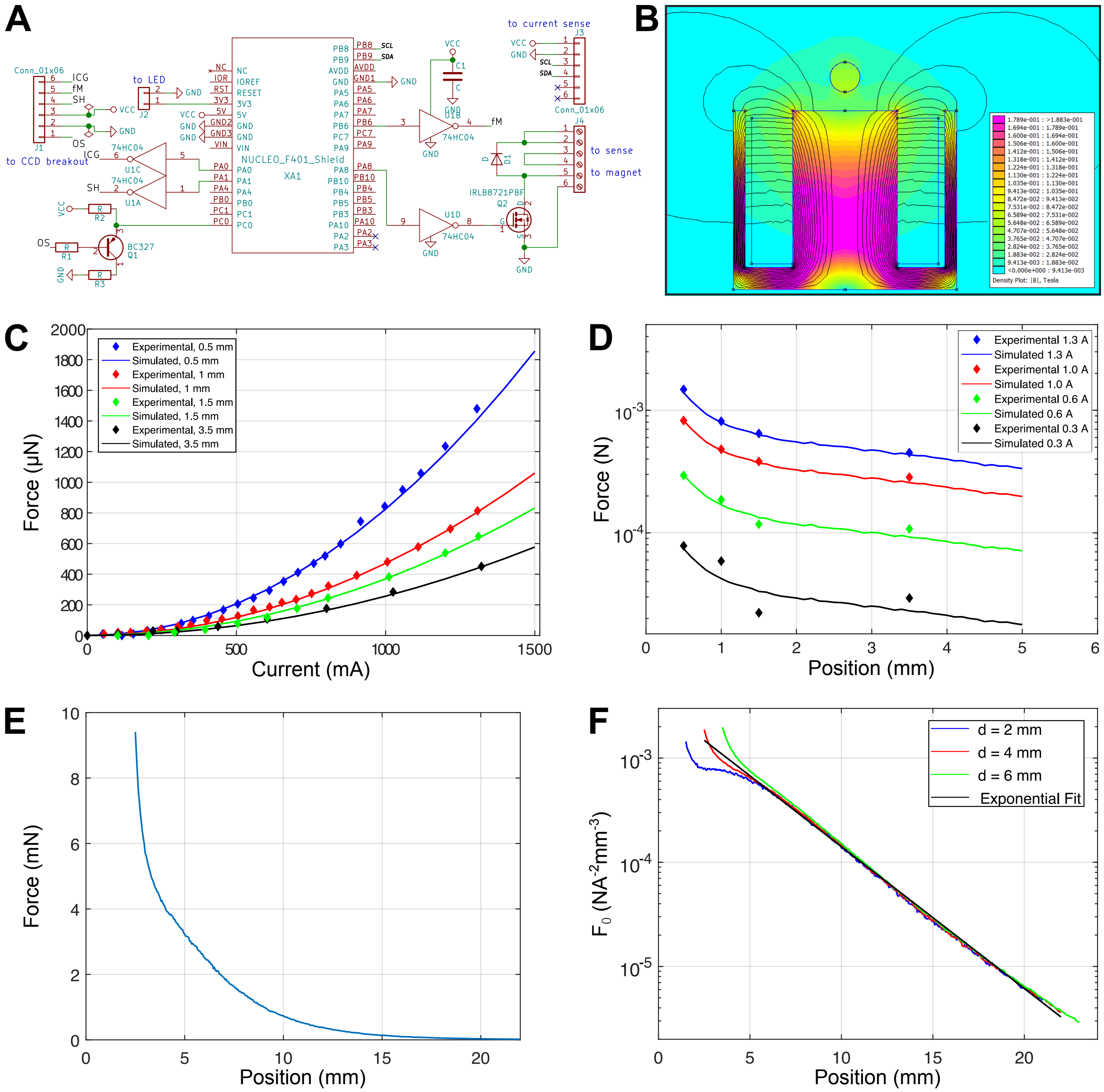

Figure S1. Characterization of the magnetic force actuator. A: Schematic of the main printed circuit board (PCB) attached to the headers of the microprocessor board, the magnet driver circuit and the breakout PCB attached to the CCD sensor. **B**: FE simulation output, showing the 2D heatmap and contour lines of the magnetic field generated by the Eclipse M52173 electromagnet in presence of a 4.0 mm diameter bead made of 1010 grade mild steel. **C**: *In silico* and *in vitro* values of the magnetic force acting on the bead against the current for a range of positions. **D**: *In silico* and *in vitro* values of magnetic force acting on the bead against its position for a range of currents. **E**: Simulated magnetic force acting on a 4.0 mm bead at I = 280.0 mA. **F**: Force coefficient, F_0 for a range of d and I values, showing exponential behavior.

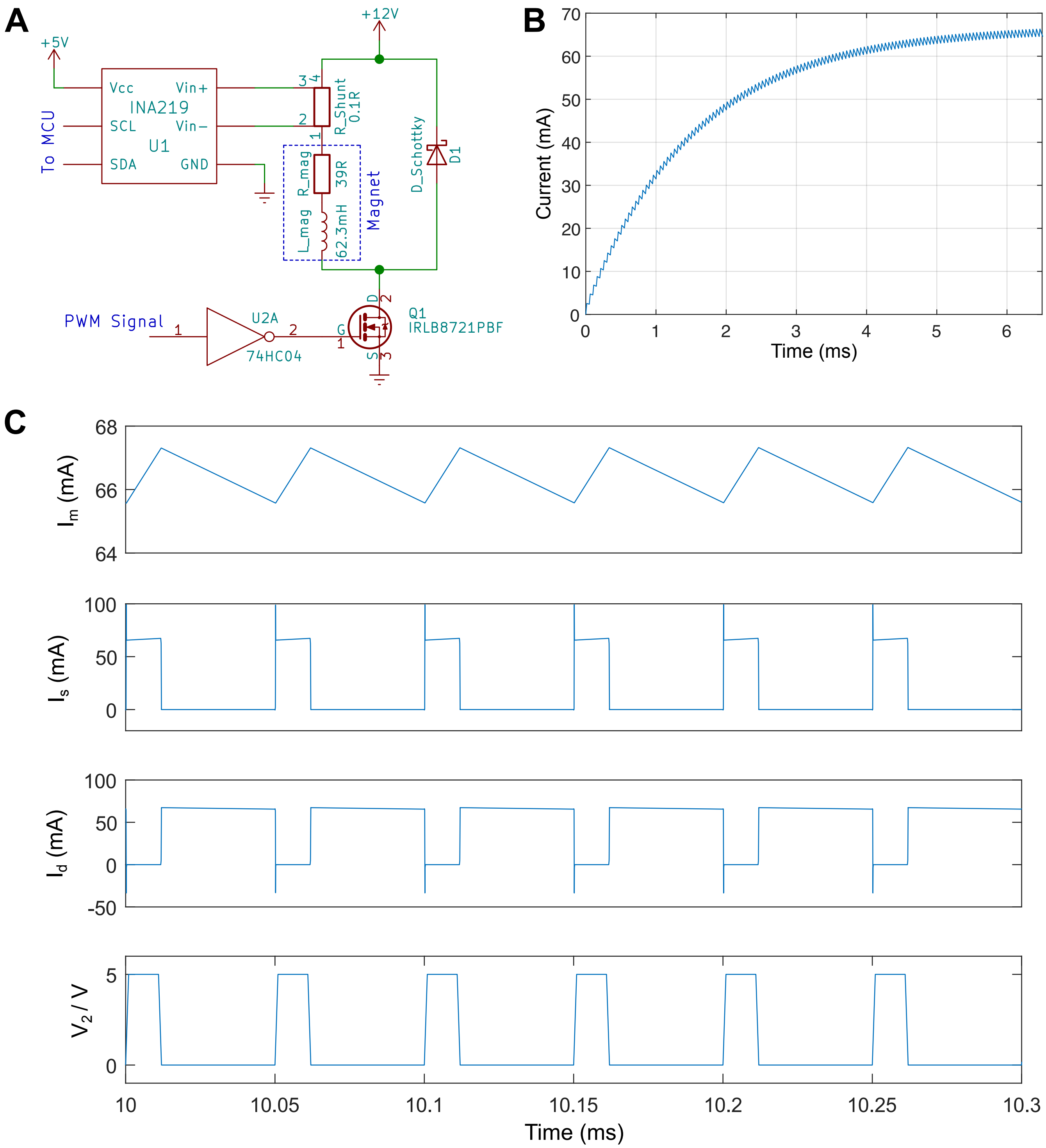

Figure S2. Design and characterization of the electromagnet driver circuit. A: Schematic of the electromagnet driver circuit. **B**: Current transient response for a step change in duty cycle. **C**: Simulated current waveforms for the electromagnet, diode and power supply (I_m, I_d, I_s) along with the driving 20% duty cycle, 20 kHz PWM signal. I_m is the sum of I_s and I_d.

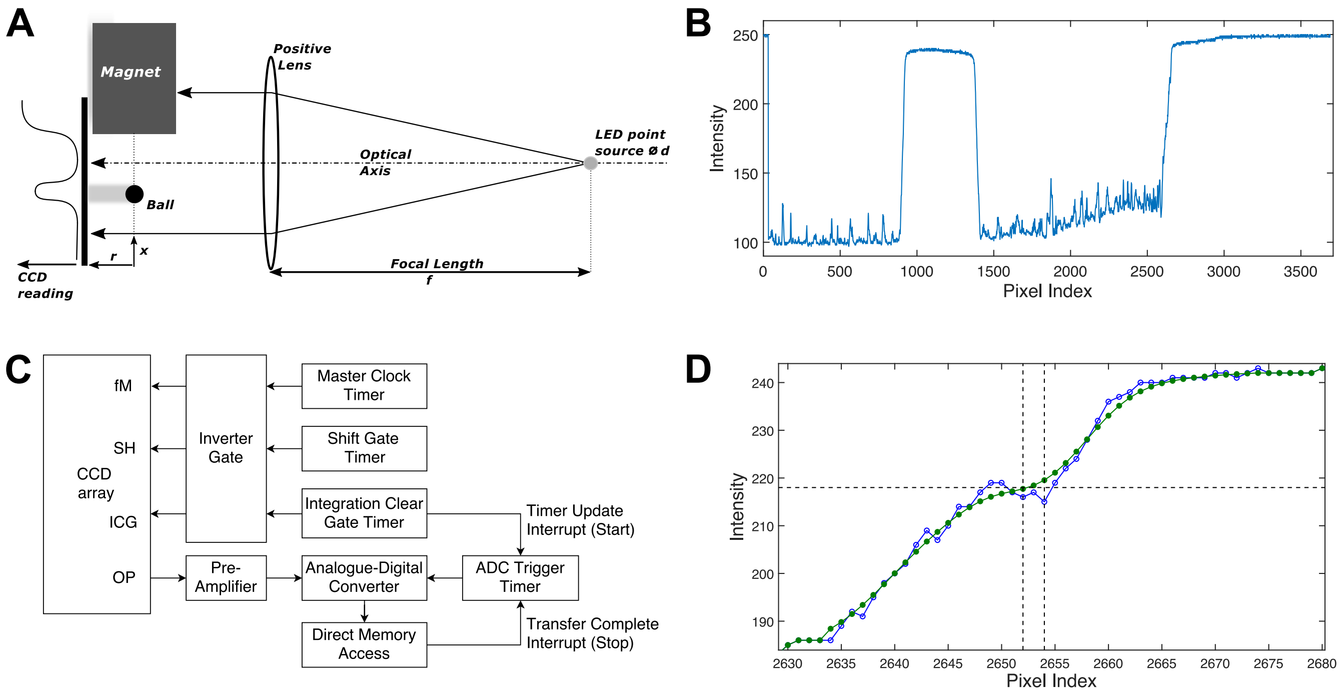

Figure S3. Design and characterization of the CCD sensor and associated embedded system. A: Schematic of the optical setup, including a lens and an LED point light source. **B**: Typical CCD output; the high intensity values correspond to dark pixels associated with the shadow. On the right, one can observe the shadow of the edge of the electromagnet and, in the region between 900 and 1400 pixels, the shadow of the steel bead and buoyancy chamber. **C**: Schematic of the CCD driver architecture. **D**: Raw (blue) and filtered (green) CCD output signals at the edge of the magnet shadow. The threshold is shown as a horizontal line, and crossing points for the raw and smoothed signals are the right and left vertical lines respectively.

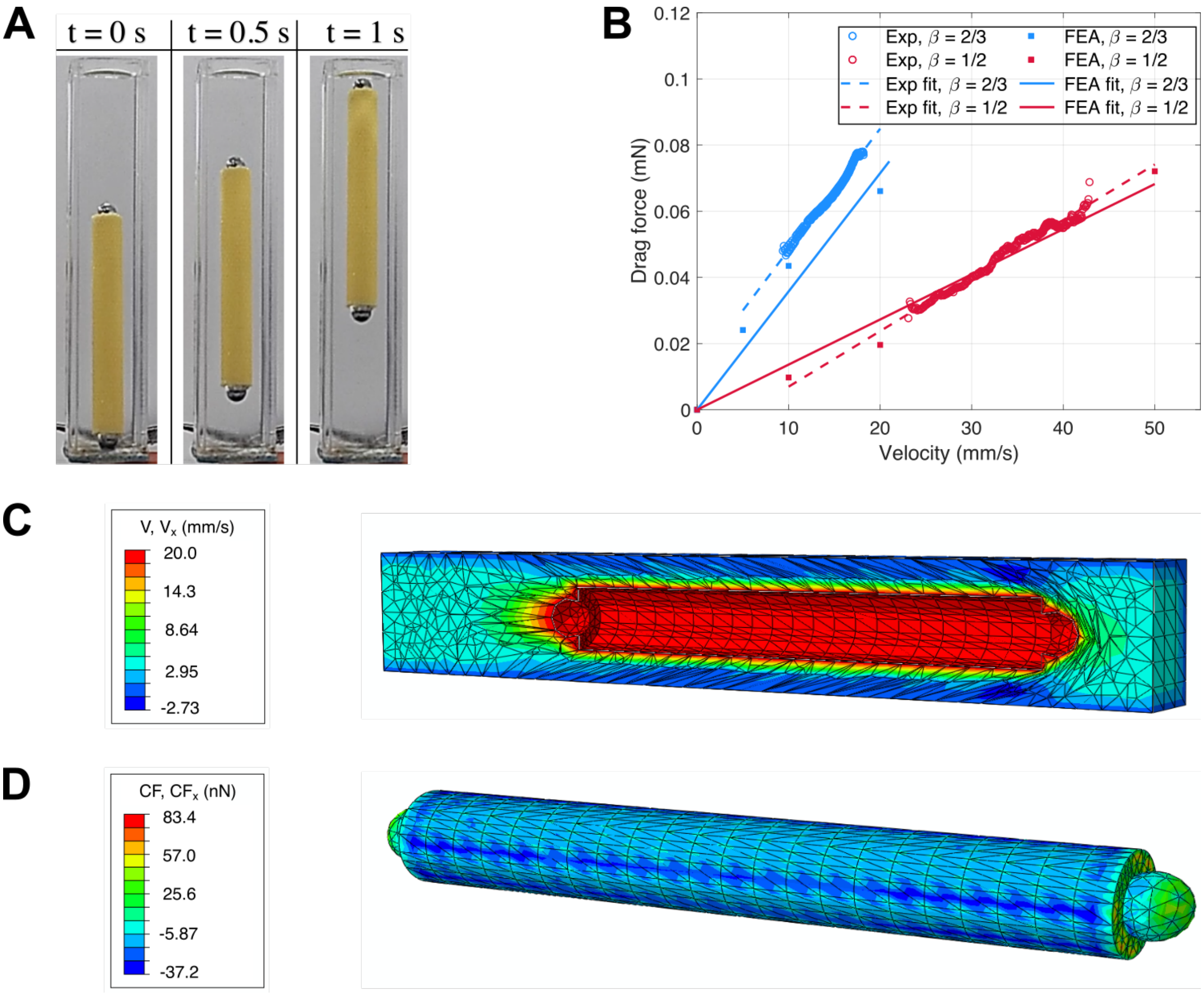

Figure S4. ***In vitro*** **and** ***in silico*** **characterization of the drag force acting on the buoyancy cell.** **A**: Experimental setup illustrating the position of the cell at t = 0, 0.5 and 1s for blockage ratio $\beta = \frac{1}{2}$. **B**: Force-velocity results for $\beta = \frac{1}{2}$ and $\beta = \frac{2}{3}$. **C**: FE results for the velocity profile for $u = 20\frac{\text{mm}}{\text{s}}$ and $\beta = \frac{2}{3}$. **D**: FE results for the concentrated force on the buoyancy cell for $u = 20\frac{\text{mm}}{\text{s}}$ and $\beta = \frac{2}{3}$.

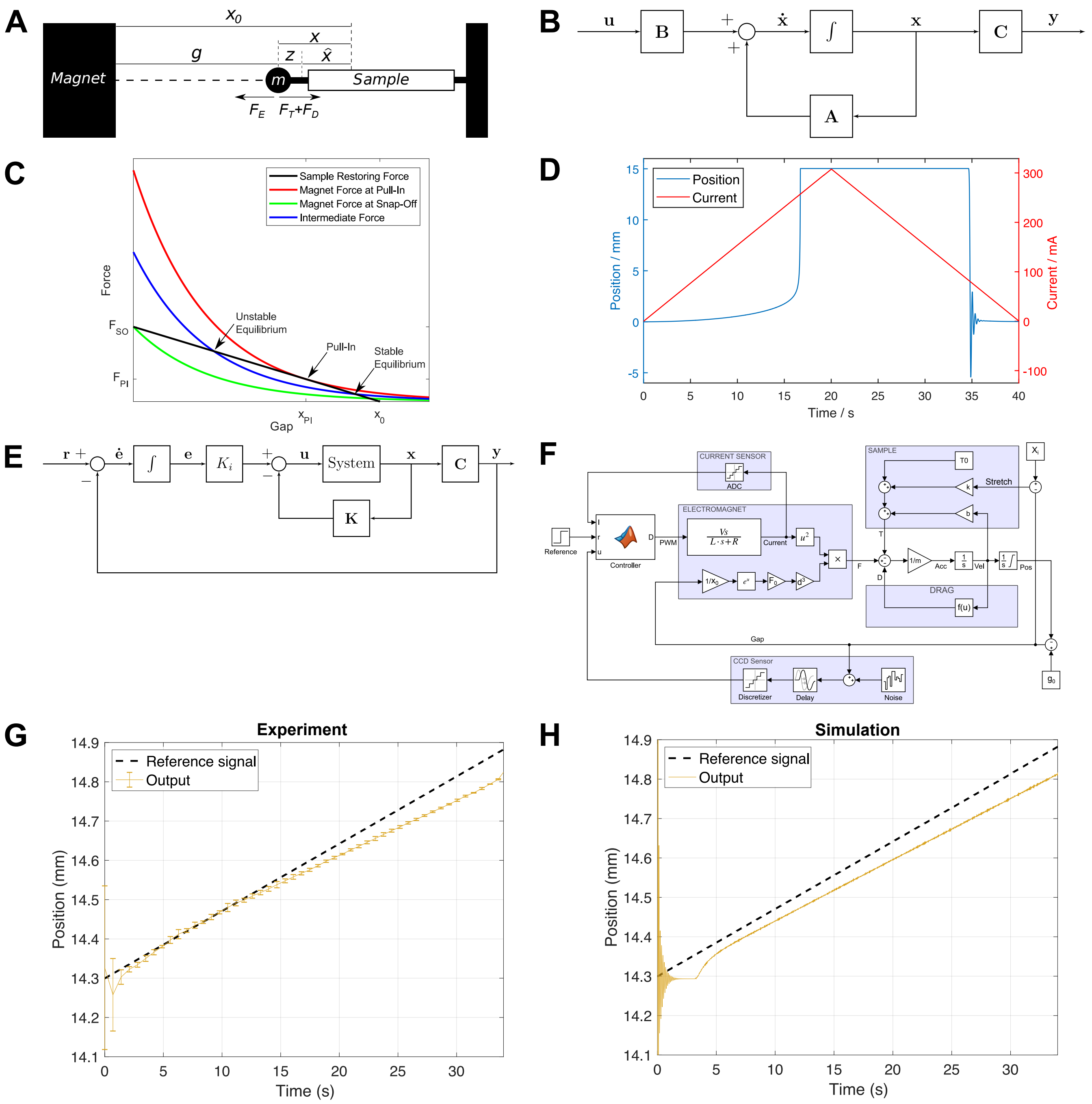

Figure S5. Mathematical model of the system and controller design. A: Schematic of the mechanical model of the system. **B**: Block diagram of the open-loop linearized system. **C**: Sample and magnet force characteristics at critical and intermediate currents. **D**: Simulation of bead pull-in and snap-off events during a current sweep. **E**: Block diagram of a state-space controller configuration with integral action. **F**: Block diagram of the SIMULINK model of the system. **G**: Experimental tracking of a reference input position signal ($n = 3$). Error bars represent 1 SD. **H**: Simulated system behavior for the same input.

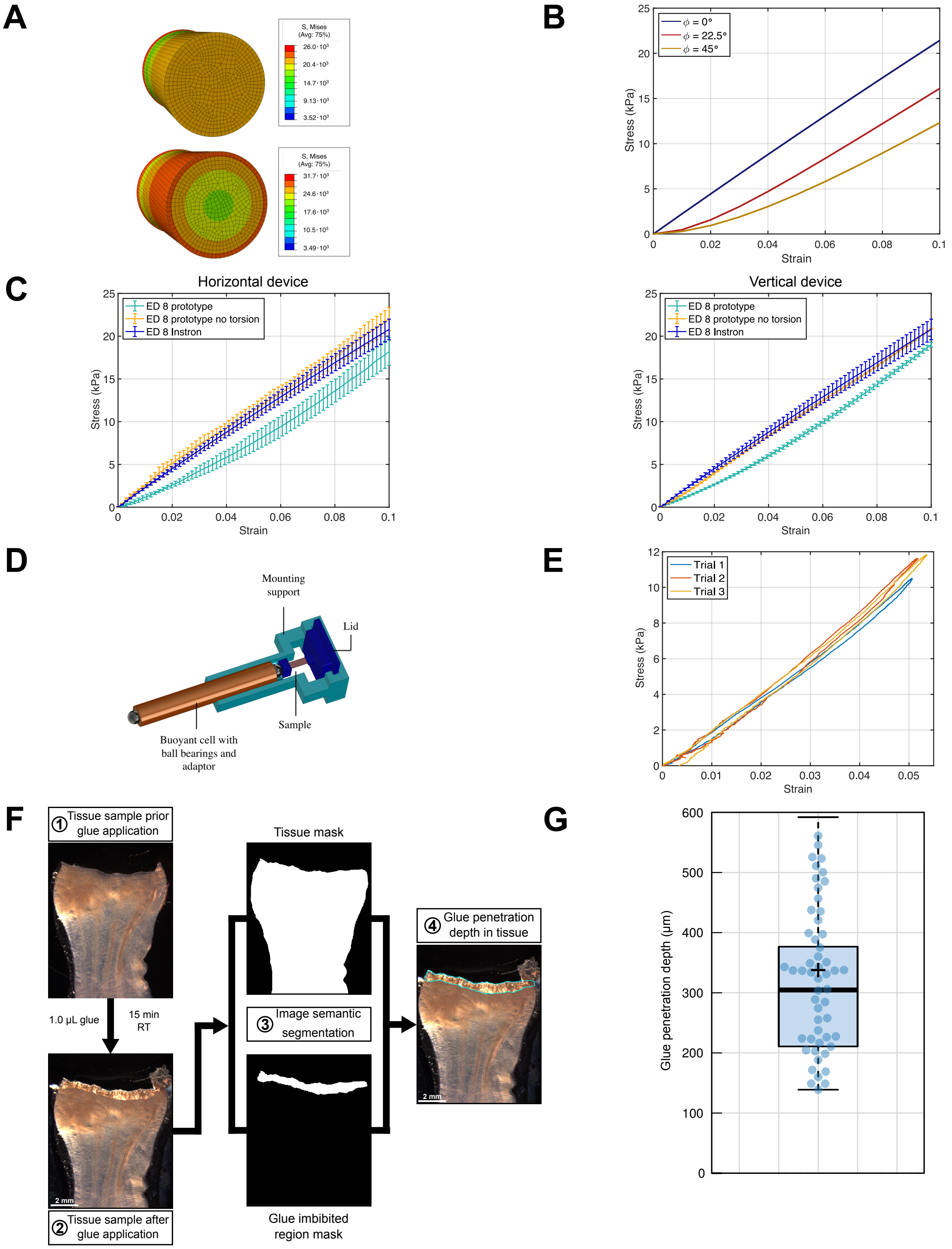

Figure S6. **Device validation: effects of sample torsion, device configuration, hysteresis, and adhesive penetration. A**: FE results showing the von Mises stress distribution at the mid-plane section for a cylindrical sample when (Top) no torsion is applied, and (Bottom) for a twisting angle of $\phi = 45°$. **B**: Stress-strain curves computed numerically for three different twisting angles ($\phi = 0°, 22.5°, 45°$). **C** (Left) stress-strain curves for Elite Double 8 samples in the horizontal device configuration (air) with and without torsion effects ($n = 3$). (Right) stress-strain curves from the vertical device configuration under the same conditions ($n = 3$). Error bars represent 1 SD. **D**: 3D model of the mounting support for sample attachment and installation into the mounting chamber. **E**: Stress-strain curves for the device in the horizontal configuration, using the mounting chamber filled with PBS. The graph shows negligible hysteresis of one Elite Double 8 sample using a triangular reference input signal. **F**: Experimental workflow for the characterization of adhesive penetration into the tissue for mounting. **G**: Adhesive penetration distribution in $n = 9$ esophageal tissue samples. Penetration was measured in six different locations for each sample.

Supplementary Information

Magnetic force actuator driver circuit

A MOSFET-based switching circuit (Fig. S2 A) was implemented to vary the input current of the electromagnet from a fixed DC voltage source. The input to the circuit is a pulse width modulated (PWM) square wave signal, which turns the MOSFET on for a fraction of the time, determined by the duty ratio D. To enable the current to flow for the fraction of the cycle when the MOSFET is off, a Schottky diode is placed parallel with the electromagnet. Measurement of the current is achieved using a $0.1\,\Omega$ high-side shunt resistor and the Texas Instruments INA 219 current sensor, connected to a microprocessor over an I2C bus. The current sensor has a 12-bit analog to digital converter, resulting in a current resolution of 0.1 mA, with a sampling period of 512 μs.

Using LTspice circuit simulation software, we evaluated the response time and the current ripple of the driving circuit as shown in Fig. S2 B-C. The response to a step change in D is consistent with that of a first-order inductor-resistor filter with a time constant $\tau = 1.6$ ms. Under typical operating conditions of 12 V and 20 kHz modulation, the ripple is limited to 2.5 mA. It is important to minimize this disturbance due to the consequent unwanted oscillatory force applied to the sample and the effect on the accuracy with which the current may be instantaneously measured. Therefore, higher switching frequencies f_{PWM} should be used, a smaller source voltage should be applied in preference to low duty cycles when testing samples that require low forces.

Optical sensor embedded system

Accurate measurements of the extension of the sample necessitate accurate estimates of the distance between the rightmost edge of the bead shadow and the leftmost edge of the magnet shadow, as shown in Fig. S3 B. This was achieved by using the pixel index at which the CCD signal drops below a certain threshold level and was calculated with high precision. Fig. S3 D shows an example where the threshold level is crossed multiple times, leading to ambiguity in the choice of critical pixel index. To increase the robustness of the measurement of the critical pixel index, we applied a low-pass smoothing filter on the raw CCD signal. The filter is hard-coded as a Gaussian window function of length 15 pixels and only applied in the signal region in the vicinity of the threshold crossing through an iterative process. Iterating through the signal from the right (decreasing pixel indexes) leads to the identification of the first crossing below the threshold value (vertical line on the right-hand side in S3 D). This provides the approximate location of the true crossing. In order to determine the precise value, the neighboring ± 20 pixels are filtered, using: $y_i = \left((W/2) + \sum_{k=0}^{k=14} w_k x_{i+k-7}\right)/W$; where x_i and y_i are the raw and filtered signals, respectively, w_k are integer filter coefficients, and W is a normalizing constant. The threshold crossing of the filtered 40-pixel signal can now be found to sub-pixel resolution using interpolation and is taken to be the true position of the magnet (the vertical line on the left-hand side in Fig. S3 D). Continuing from the leftmost end of the filtered region, the same algorithm may be used to calculate the exact location of the right edge of the buoyancy cell and bead shadow, by identifying the point at which the filtered signal exceeds the threshold again. The speed of this algorithm was optimized from an execution time of around 1.5 to 0.4 ms by adding a further coarse searching step, whereby every 50th pixel is sampled to identify the 50-pixel block the transition lies in before applying the original algorithm to that block.

Mathematical model of the system

The dynamics of the system is governed by Newton's Second Law, whereby the interaction between the electromagnet and the ferromagnetic bead is opposed by the tension in the sample and the fluid resistance (Fig. S5 A), as described in equations 1 and 2:

$$\sum \vec{F} = m\vec{a} \tag{1}$$

$$F_{\mathrm{E}} - (F_{\mathrm{T}} + F_{\mathrm{D}}) = ma \tag{2}$$

where E, T, and D refer to the electromagnetic, tensile, and drag forces respectively. This can be expressed in the standard state-space form:

$$\dot{\mathbf{x}} = \mathbf{f}(\mathbf{x}, \mathbf{u}) \tag{3}$$

where $\mathbf{x}$ and $\mathbf{u}$ are the state and input vectors respectively, and $\mathbf{f}$ is the state transition function. Using equation 2, and recalling that the input to the system is the current I driving the electromagnet, $\mathbf{x}$ and $\mathbf{u}$ can be rewritten as:

$$\mathbf{x} = \begin{bmatrix} \dot{x} \\ \ddot{x} \end{bmatrix} = \begin{bmatrix} \dot{x} \\ \frac{F_{\mathrm{E}} - (F_{\mathrm{T}} + F_{\mathrm{D}})}{m} \end{bmatrix} = \begin{bmatrix} \dot{x} \\ \frac{f_{\mathrm{E}}(I, x - x_0) - (f_{\mathrm{T}}(x, \dot{x}) + f_{\mathrm{D}}(\dot{x}))}{m} \end{bmatrix} \tag{4}$$

$$\mathbf{u} = I^2 \tag{5}$$

Rewriting this system in the standard linear form, as shown in the block diagram in Fig. S5 B, yields:

$$\begin{cases} \dot{\mathbf{x}} = \mathbf{A}\mathbf{x} + \mathbf{B}\mathbf{u} \\ \mathbf{y} = \mathbf{C}\mathbf{x} \end{cases} \tag{6}$$

where the output of the system $\mathbf{y}$ is the position of the ferromagnetic bead measured by means of the optical sensor and it is related to the state vector $\mathbf{x}$ by the matrix $\mathbf{C}$:

$$\mathbf{C} = \begin{bmatrix} 0 & 1 \end{bmatrix} \tag{7}$$

In order to compute matrices $\mathbf{A}$ and $\mathbf{B}$, the system must be linearized in the vicinity of the equilibrium point $\hat{x}$:

$$\begin{cases} z = x - \hat{x} \\ u = I^2 - \hat{I}^2 \end{cases} \tag{8}$$

Computation of the first order Taylor expansion of $\mathbf{f}$ yields in the Jacobian matrix form:

$$\begin{aligned} \begin{bmatrix} \dot{z} \\ \ddot{z} \end{bmatrix} &= \begin{bmatrix} \frac{\partial f_1}{\partial z} & \frac{\partial f_1}{\partial \dot{z}} \\ \frac{\partial f_2}{\partial z} & \frac{\partial f_2}{\partial \dot{z}} \end{bmatrix}\Bigg|_{\substack{\mathbf{x}=\hat{\mathbf{x}} \\ \mathbf{u}=0}} \begin{bmatrix} z \\ \dot{z} \end{bmatrix} + \begin{bmatrix} \frac{\partial f_1}{\partial u} \\ \frac{\partial f_2}{\partial u} \end{bmatrix}\Bigg|_{\substack{\mathbf{x}=\hat{\mathbf{x}} \\ \mathbf{u}=0}} \begin{bmatrix} u \end{bmatrix} \\ &= \underbrace{\begin{bmatrix} 0 & 1 \\ \frac{1}{m}\left(\frac{\partial f_{\mathrm{E}}}{\partial x} - \frac{\partial f_{\mathrm{T}}}{\partial x} - \frac{\partial f_{\mathrm{D}}}{\partial x}\right) & -\frac{1}{m}\left(\frac{\partial f_{\mathrm{T}}}{\partial \dot{x}} + \frac{\partial f_{\mathrm{D}}}{\partial \dot{x}}\right) \end{bmatrix}\Bigg|_{\substack{\mathbf{x}=\hat{\mathbf{x}} \\ \mathbf{u}=0}}}_{\mathbf{A}} \begin{bmatrix} z \\ \dot{z} \end{bmatrix} + \underbrace{\begin{bmatrix} 0 \\ \frac{1}{m}\frac{\partial f_{\mathrm{E}}}{\partial I^2} \end{bmatrix}\Bigg|_{\substack{\mathbf{x}=\hat{\mathbf{x}} \\ \mathbf{u}=0}}}_{\mathbf{B}} \begin{bmatrix} u \end{bmatrix} \end{aligned} \tag{9}$$

The coefficients of matrix $\mathbf{B}$ are only defined by the characteristics of the magnetic interaction between the bead and the electromagnet, whereas $\mathbf{A}$ depends primarily on the tensile force generated by the test specimen. To derive the tensile force, we used a Kelvin-Voigt model consisting of a spring of stiffness, k, and a dashpot of viscosity, b, in parallel. We also considered a constant force term T_0 representing the weight component along the tensile direction:

$$F_{\mathrm{T}} = T_0 + kx + b\dot{x} \tag{10}$$

Rewriting the partial derivatives in equation 9:

$$\frac{\partial f_{\mathrm{E}}}{\partial x} = k_x \qquad \frac{\partial f_{\mathrm{E}}}{\partial I^2} = k_I \tag{11}$$

$$\frac{\partial f_{\mathrm{T}}}{\partial x} = k \qquad \frac{\partial f_{\mathrm{T}}}{\partial \dot{x}} = b \tag{12}$$

$$\frac{\partial f_{\mathrm{D}}}{\partial x} = 0 \qquad \frac{\partial f_{\mathrm{D}}}{\partial \dot{x}} = \mu \tag{13}$$

we derived an analytical expression for matrices $\mathbf{A}$ and $\mathbf{B}$:

$$\mathbf{A} = \begin{bmatrix} 0 & 1 \\ \frac{k_x - k}{m} & -\frac{b+\mu}{m} \end{bmatrix} \qquad \mathbf{B} = \begin{bmatrix} 0 \\ \frac{k_I}{m} \end{bmatrix} \tag{14}$$

Here, k and b are the parameters of the Kelvin-Voigt model, k_x and k_I are the so-called magnetic spring constants, and μ is fluid viscosity constant. The stability of the open-loop system can now be assessed in deriving its poles from the characteristic equation (i.e. the eigenvalues of $\mathbf{A}$):

$$\det(\lambda\mathbf{I} - \mathbf{A}) = \lambda^2 + \frac{b+\mu}{m}\lambda + \frac{k_x - k}{m} \tag{15}$$

According to the Routh-Hurwitz criterion, stability is achieved when the poles have negative real parts equivalent to the coefficients of the positive characteristic equation. Thus, without use of feedback loop, the system would be prone to instability for any $k_x > k$.

As shown in Fig. S5 C, the equilibrium points of the open-loop system are reached when the magnet force balances the sample restoring force ($F_E = F_T$), assuming the drag force to be negligible. Considering the point of stable equilibrium, where $k > k_x$, a small deviation in bead position to the left leads to a greater increase in restoring force than in magnet force. The bead thus returns towards equilibrium. By contrast, a deviation to the left at the point of unstable equilibrium leads to a greater increase in magnet force compared to the restoring force. The bead will therefore continue to accelerate towards the magnet. As the current is increased, a limiting case occurs when $F_T(x)$ becomes tangential to $F_E(x)$ with k_x and k being equal. Beyond this marginally stable case, there is no equilibrium position at which the sample tension is equal to the magnet force, and the bead accelerates towards the magnet, an event termed pull-in. Stability cannot be achieved at positions greater than the pull-in location. Reducing the current below the pull-in value does not immediately release the bead; rather, the current must be reduced until the green line in Fig. S5 C is reached. At this point, the bead accelerates towards the stable position near x_0, an event termed snap-off. This behavior is evidenced by the simulation in Fig. S5 D, with the current at pull-in being much greater than at snap-off (260 and 80 mA, respectively).

Controller design

Analysis of the open-loop system clearly emphasizes the need for a controller and closed-loop feedback. Without feedback, pull-in may occur at low elongations, severely limiting the operating range of the instrument. To overcome this limitation, a PID controller was implemented. As shown in Fig. S5 E, the controller takes as input the state vector **x** and the error between the reference **r** and the output **y**. Integration of the error with respect to time results in an internal integrator state **e** and the control signal **u**, which can be calculated:

$$\dot{\mathbf{e}} = \mathbf{r} - \mathbf{y} = \mathbf{r} - \mathbf{C}\mathbf{x} \tag{16}$$

$$\mathbf{u} = K_i\mathbf{e} - \mathbf{K}\mathbf{x} = K_i\mathbf{e} - \begin{bmatrix} K_p & K_d \end{bmatrix} \begin{bmatrix} z \\ \dot{z} \end{bmatrix} \tag{17}$$

where K_p, K_i, and K_d are, in order, the proportional, the integral, and the derivative gains. The proportional gain acts to modulate the current in opposition to the deviation of the ferromagnetic bead from the desired position; thus, if the bead moves closer to the electromagnet by, the square of the current is reduced by an amount K_p, allowing the bead to return towards equilibrium. The role of the derivative term is to add damping to the response by opposing the velocity of the bead, while the integrator acts to prevent steady state error in the equilibrium position. Here, the state-space representation of the system becomes:

$$\begin{bmatrix} \dot{\mathbf{x}} \\ \dot{\mathbf{e}} \end{bmatrix} = \begin{bmatrix} \mathbf{A} & 0 \\ -\mathbf{C} & 0 \end{bmatrix} \begin{bmatrix} \mathbf{x} \\ \mathbf{e} \end{bmatrix} + \begin{bmatrix} \mathbf{B} \\ 0 \end{bmatrix} \mathbf{u} + \begin{bmatrix} 0 \\ \mathbf{I} \end{bmatrix} \mathbf{r} \tag{18}$$

From combination of equations 17 and 18, the closed-loop state transition matrix $\mathbf{A}_{CL}$ can be defined as:

$$\mathbf{A}_{CL} = \frac{1}{m} \begin{bmatrix} 0 & m & 0 \\ (k_x - k - k_I K_p) & (-b - \mu - k_I K_d) & k_I K_i \\ -m & 0 & 0 \end{bmatrix} \tag{19}$$

The poles of the closed-loop system are the eigenvalues of $\mathbf{A}_{CL}$, i.e. the roots of the characteristic polynomial below:

$$\det(\lambda I - \mathbf{A}_{CL}) = \frac{1}{m}(m\lambda^3 + (b + \mu + k_I K_d)\lambda^2 + (k + k_I K_p - k_x)\lambda + k_I K_i) \tag{20}$$

To compute the gains of the PID controller, this expression can be used in combination with the desired characteristic equation. This is obtained upon the choice of the closed-loop poles p, as follows:

$$\begin{aligned} &(\lambda - p_1)(\lambda - p_2)(\lambda - p_3) \\ &= \lambda^3 - (p_1 + p_2 + p_3)\lambda^2 + (p_1p_2 + p_1p_3 + p_2p_3)\lambda + p_1p_2p_3 \\ &= \lambda^3 + \alpha_0\lambda^2 + \alpha_1\lambda + \alpha_2 \end{aligned} \tag{21}$$

Therefore, the controller gains can be calculated by matching the coefficients of equations 20 and 21. Assuming that k, b, and μ are zero in the first iteration:

$$K_p = \frac{k_x + m\alpha_1}{k_I} \qquad K_d = \frac{m\alpha_0}{k_I} \qquad K_i = \frac{m\alpha_2}{k_I} \tag{22}$$

Device simulation

As shown in Fig. S5 F, the SIMULINK model consists of six main components, namely the *electromagnet*, the *sample*, the *drag*, the *current sensor*, the *CCD sensor*, and the *controller*:

Electromagnet: The *electromagnet* receives as inputs the distance between the metallic bead and the electromagnet and the duty cycle D of the PWM signal from the controller. This allows the calculation of the current from:

$$\frac{\bar{I}(s)}{\bar{D}(s)} = \frac{V_{\mathrm{s}}}{Ls+R} \tag{23}$$

where V_{s} is the supplied voltage, and L and R correspond to the inductance and resistance of the circuit, respectively. Thus, given a measure of the electric current, the electromagnetic force can be computed.

Sample: Integration of the acceleration, calculated as per equation 2, allows computation of the velocity and of the position of the bead. The elongation of the sample is therefore obtained, at each iteration, via comparison of the distance between the bead and the electromagnet with its initial value x_{i}. The velocity and the elongation of the specimen are then multiplied by the viscosity and stiffness material constants, respectively, for calculation of the generated tension as per equation 10.

Drag: The bead velocity is utilized to compute the drag force. The combination of the electromagnetic force with the sample tension and drag resistance yields the net force acting on the sample (equation 2).

Current and CCD sensors: The current and image sensors provide inputs to the PID controller. The former measures the current from the *Electromagnet* as per equation 23, whereas the latter records the distance between the bead and the electromagnet and is modeled to simulate noise and delay added to the system.

Controller: Alongside the inputs from the current and image sensors, the PID controller receives as inputs the reference position to be tracked, generally modeled as a ramp or a triangular signal. The output of this block is the duty cycle, which is in turn fed to the *Electromagnet* block for the following iteration to begin.

Modelling soft biological tissues as composite materials

To predict the mechanical behavior of multi-layered biological tissues or organs, given the response of each individual tissue layer, we used generalization of the *Rule of mixtures*, traditionally adopted to describe the behavior of composite materials. Our formulation is based on the analogous assumptions of ideal inter-layer bonding and uniaxial loading, while accounting for the non-zero residual stresses and strains of each individual layer. Nevertheless, it is valid exclusively in the regions of the stress-strain curve where the tissue exhibits an approximately linear elastic response.

We modeled a representative volume element (RVE) of the esophageal wall as composed of epithelial (E), stromal (S), and muscle (M) layers. These are of variable length l and constant cross-sectional area A. When stress $\sigma = \frac{F}{A}$ is applied to the RVE in the longitudinal direction, strain ε is generated as per $\varepsilon = \frac{l-l_0}{l_0}$. Generalizing for i = 1 to N layers, from the principle of static equilibrium, we obtain:

$$\sigma_P A_P = \sum_{i=1}^{N} \sigma_i A_i \tag{24}$$

where the subscript P refers to the intact physiological state. The left- and right-hand sides of the equation represent the intact and the zero-stress states, respectively. For equation 24 to hold, the physiological non-zero residual stress σ_R of each layer must be considered together with the stress due to stretching σ_S, yielding:

$$\sigma_P A_P = \sum_{i=1}^{N} (\sigma_{Ri} + \sigma_{Si}) A_i \tag{25}$$

Dividing each side by A_P, and for constant width, this can be rewritten as:

$$\sigma_P = \sum_{i=1}^{N} (\sigma_{Ri} + \sigma_{Si})\, \tau_i \tag{26}$$

where τ_i is the thickness fraction of each layer with respect to the intact RVE, such as $\sum_{i=1}^{N} \tau_i = 1$. Thus, taking the first derivative of the stress with respect to strain, and considering non-zero residual strains on the right-hand side, we can derive an expression of the Young's modulus at physiological strains:

$$E_P = \left.\frac{d\sigma_P}{d\varepsilon}\right|_{\varepsilon=\varepsilon_P} = \left.\sum_{i=1}^{N} \frac{d(\sigma_{Ri} + \sigma_{Si})}{d\varepsilon} \tau_i \right|_{\varepsilon_P=\varepsilon_{Ri}+\varepsilon_{Si}} \tag{27}$$

For constant values of σ_R, this expression can be reduced to:

$$E_P = \left.\sum_{i=1}^{N} \frac{d\sigma_{Si}}{d\varepsilon} \tau_i \right|_{\varepsilon_P=\varepsilon_{Ri}+\varepsilon_{Si}} = \sum_{i=1}^{N} E_{Pi} \tau_i \tag{28}$$

Equation 28 relates the Young's modulus (E_P) of the intact esophageal wall with that of each constitutive tissue layer in the zero-stress state, E_{Pi}, determined at $\varepsilon_P = \varepsilon_{Ri} + \varepsilon_{Si}$, where ε_{Ri} is the residual strain and ε_{Si} are the strain applied during tensile testing. This formulation represents a weighted sum of the contribution of each tissue layer, whereby the calculation of E_{Pi} is shifted by an amount equal to the residual strain of each individual layer.
